# Comprehensive Genome-Wide Exploration of C2H2 Zinc Finger Family in Grapevine (*Vitis vinifera* L.): Insights into the Roles in the Pollen Development Regulation

**DOI:** 10.3390/genes12020302

**Published:** 2021-02-20

**Authors:** Oscar Arrey-Salas, José Carlos Caris-Maldonado, Bairon Hernández-Rojas, Enrique Gonzalez

**Affiliations:** 1 Laboratorio de Genómica Funcional, Instituto de Ciencias Biológicas, Universidad de Talca, 3460000 Talca, Chile; egonzale@utalca.cl; 2 Center for Research and Innovation (CRI), Viña Concha y Toro, Ruta k-650 km 10, 3550000 Pencahue, Chile; jose.caris@conchaytoro.cl; 3 Ph.D Program in Sciences Mention in Modeling of Chemical and Biological Systems, Faculty of Engineering, University of Talca, Calle 1 Poniente, 1141, 3462227 Talca, Chile; bhernandez@utalca.cl

**Keywords:** grapevine (*Vitis vinifera* L.), C2H2 zinc-finger protein, transcription factor, genome-wide, gene expression profiling, pollen development

## Abstract

Some C2H2 zinc-finger proteins (ZFP) transcription factors are involved in the development of pollen in plants. In grapevine (*Vitis vinifera* L.), it has been suggested that abnormalities in pollen development lead to the phenomenon called parthenocarpy that occurs in some varieties of this cultivar. At present, a network involving several transcription factors types has been revealed and key roles have been assigned to members of the C2H2 zinc-finger proteins (ZFP) family in model plants. However, particularities of the regulatory mechanisms controlling pollen formation in grapevine remain unknown. In order to gain insight into the participation of ZFPs in grapevine gametophyte development, we performed a genome-wide identification and characterization of genes encoding ZFP (*VviZFP* family). A total of 98 genes were identified and renamed based on the gene distribution into grapevine genome. The analysis performed indicate significant changes throughout *VviZFP* genes evolution explained by high heterogeneity in sequence, length, number of ZF and presence of another conserved domains. Moreover, segmental duplication participated in the gene family expansion in grapevine. The *VviZFPs* were classified based on domain and phylogenetic analysis into three sets and different groups. Heat-map demonstrated differential and tissue-specific expression patterns of these genes and k-means clustering allowed to identify a group of putative orthologs to some ZFPs related to pollen development. In transgenic plants carrying the promVviZFP13::GUS and promVviZFP68::GUS constructs, GUS signals were detectable in the anther and mature pollen grains. Expression profiling of selected *VviZFP* genes showed differential expression pattern during flower development and provides a basis for deepening in the understanding of *VviZFPs* role on grapevine reproductive development.

## 1. Introduction

Male reproductive development in plants is a highly regulated process where transcription factors (TF) have been shown to play a fundamental role. In Arabidopsis (*Arabidopsis thaliana* (L.) Heynh) [1], the most abundant family of transcriptional regulators contains one or more zinc-finger domains (ZF) and, for this reason, they are named zinc-finger proteins (ZFP). In the ZF domain, a zinc ion is coordinated by cysteine (C) and/or histidine (H) residues conforming a finger type three-dimensional structure. The ZFPs are classified according to the number and location of C and H residues [2]. C2H2-ZFPs, which are otherwise termed as TF3A-type ZFP or the Krüppel-like ZFPs, can interact with DNA, RNA, and proteins, participating not only in transcriptional regulation but also in chromatin regulation and RNA metabolism [3]. This versatility is expressed in the diversity of biological functions performed by these proteins.

There is a subfamily of C2H2-ZFPs named Q-type C2H2-ZFP that contain an invariant QALGGH motif within the putative DNA-contacting surface in the ZF domain. This motif has not been reported in other organisms besides plants, suggesting that this protein group may be regulating unique processes in plant life [4].

Since the first C2H2-ZFP was cloned in 1992 [5], the role of these proteins in different physiological and development processes has been investigated [3,6,7]. These results have allowed associating some of them with anther and pollen development [3]. For example, in petunia (*Petunia* × *atkinsiana* (Sweet) D.Don ex W.H.Baxter), seven *ZFPs* genes (*PhZPT*) are sequentially expressed along anther development [8]. The earliest expressed gene, *ZPT3-2*, renamed *TAPETUM DEVELOPMENT ZINC FINGER PROTEIN 1* (*TAZ1*), is specifically transcribed in tapetum after pollen mother cells (PMC) meiosis. Silencing *TAZ1* expression resulted in aberrant development and premature degeneration of the tapetum, abortion of the microspore and a limited production of pollen grains, which also showed defects in the pollen wall [9]. The second expressed gene, *ZPT2-5*, renamed as *MEIOSIS-ASSOCIATED ZINC-FINGER PROTEIN 1* (*MEZ1*), is first specifically transcribed in PMC during the early stages of anther development and later in vegetative tissues. Silencing *MEZ1* resulted in several anomalies during male meiosis by forming an abnormal number of meiocytes with varying DNA contents [10]. The role of the other *PhZPT* genes in this process remains to be elucidated. In *Brassica campestris*, the *BcMF20* gene, homologue of the arabidopsis gene *At1g26610*, is specifically expressed during the development of pollen grains and tapetum but the assignment of its specific role is unknown [11]. In arabidopsis during microspore development, the role of a germline-specific MYB transcription factor (DUO1) is particularly interesting since it activates the target genes *DUO1-ACTIVATED ZINC FINGER 1* (*DAZ1*) and *DAZ2*, two Q-type *ZFP* genes. These redundant genes are required for germ cell division and sperm cells differentiation as well for gamete fusion at fertilization. In the proposed model, DAZ1 and DAZ2 promote cell division and differentiation by repressing an unknown repressor of both processes in an EAR motif-dependent mode [12].

A subset of ZFPs have been identified as active repressors in various plant species, including arabidopsis, wheat (*Triticum aestivum* L.), petunia, and soybean (*Glycine max* (L.) Merr.). The repression motif usually is located in their C-terminal region, designated as the ERF-associated amphiphilic repression (EAR) motif and contains a conserved consensus sequence of ^L^/_F_DLN^L^/_F_(x)P [13]. Sequence comparison of the core EAR motif sites from these proteins in arabidopsis [14] revealed two distinct conservation patterns: LxLxL (X can be one of the 20 common amino acids) containing three conserved Leu residues in alternate positions, and DLNxxP, consisting of a conserved DLN box and a Pro residue at the fifth or sixth position. The Asp and Leu residues have been shown to be implicated in repression activity [13,15,16]. The deletion of EAR-motif regions of ZINC FINGER OF ARABIDOPSIS 10 (ZAT10) [17], ZAT11 [18], DAZ1 and DAZ2 [12] eliminated the capability to repress transcription, supporting the importance of EAR-motif in the role of these ZFPs.

Grapevine (*Vitis vinifera* L.) is one of the world’s most important fruit crops with highly valued berries used for products such as juices and wines. Some cultivars (ie. Carménère, Malbec and Merlot) exhibit a high tendency to have parthenocarpic fruit development (PFD) [19], a developmental disorder that seriously affects quality traits. PFD is characterized by the presence in the same bunch of normal size seeded berries together with medium-sized, seedless and incompletely-ripened berries [19]. Different reasons, for example, vine vigor status (also considering rootstocks), pest and disease incidence, adverse weather conditions over the flowering period (extreme temperatures and water deficit stress) and deficiency in essential micronutrients such as Boron (B) or zinc (Zn) [19,20] have been associated with PFD. Zn is required as a cofactor for over 300 enzymes and proteins involved in cell division, nucleic acid metabolism and protein synthesis, and is critical in the control of gene transcription and the coordination of other biological processes regulated by proteins containing DNA-binding Zinc-finger motifs, among others the C2H2 ZFP [21,22]. Pollen germination capability is fundamental for fertility. In some grapevine cultivars, the presence of normal pollen together with anomalous grains (abnormal number of apertures or collapsed) determine a high reduction in productivity [23,24]. Alva et al. 2015 showed that in the grapevine cultivars evaluated, the PFD rate shows a straight correlation with the abnormal pollen rate, suggesting that the defective ovule fertilization could be caused by morphologically abnormal pollen that possess a reduced germination capability or the inability to elongate the pollen tube [19,25]. In this context, identifying the *ZFP* genes that are related to the transcriptional regulation of pollen formation in grapevine should be the first to be elucidated. However, so far, there is no information about the role of ZFPs in grapevine nor their possible participation integrating the pollen formation regulation pathways. Therefore, this work reports the genome-wide identification of 98 C2H2-ZFP in the grapevine genome together with an analysis of the chromosomal location, intron-exon organization, conserved domains in protein primary structure and phylogenetic relationships. In addition, a global gene expression profile was created for the different tissues and developmental stages with a special focus on pollen development. Finally, qPCR analysis and GUS histochemical staining of selected VviZFP genes were performed. Thus, this work provides a foundation for future studies in the *VviZFP* gene family evolution as well as the characterization of candidate genes related to pollen development regulation, which could contribute to finding the molecular bases that give an explanation to PFD in the varieties of Camenere, Merlot and Malbec.

## 2. Materials and Methods 

### 2.1. Identification of C2H2-Type ZF Transcription Factors in Grapevine

The genomic, coding and protein *V. vinifera* sequences were downloaded from the 12X.v2 version (and VCost.v3 annotation version) of the grapevine genome assembly [26] hosted at the URGI research unit (https://urgi.versailles.inra.fr/Species/Vitis (accessed on 19 February 2021)). The identification of C2H2-type zinc-finger proteins (VviZFPs) was made using the grapevine deduced proteome using the HMMer package v3.1b2 [27] and the Pfam domain ZF-C2H2 HMM profile (PF00096) [28]. Then, all the protein sequences obtained were subjected to domain analyses using SMART [29] and InterProScan [30] softwares with default parameters. Those protein sequences lacking the C2H2 ZF domain were discarded.

### 2.2. Determination of Chromosomal Location and Gene Structure

The *VviZFP* encoded gene locations on grape chromosomes were obtained from the annotation GFF3 file. The genes were renamed from *VviZFP1* to *VviZFP98* based on their distributions and relative linear orders among the respective chromosomes. The R package RIdeogram version 0.2.2 [31] was used to map and visualize genes in chromosomes. The Gene Structure Display Server software 2.0 [32] was used to illustrate the *VviZFPs* exon/intron organization by aligning the CDS with their corresponding genomic sequences.

### 2.3. Phylogenetic Analysis and Identification of Conserved Motifs in VviZFP Protein Sequences

Multiple protein sequence alignments were done by using the Clustal Omega program with default parameters [33]. Phylogenetic analysis was performed with MEGA X software (version 10.2.2) [34] by the Neighbor-Joining method [35] and the reliability of branching was assessed by the bootstrap resampling method using 1000 replications. The subcellular localization prediction was made in CELLO v2.5 software [36] and the prediction of nuclear localization signals (NLSs) specific to the importin αβ-dependent pathway was made in cNLS mapper [37] by filtering those with a score ≥7. The detection of conserved motifs between the ZF domains was made with “Multiple Expectation Maximization for Motif Elicitation”tool (MEME) [38] and the strategy for the identification and classification of EAR motif-containing VviZFPs was reported in [14]. The molecular weight and theoretical pI were predicted by ProtParam tool [39]. Finally, the VviZFPs were classified in the A, B or C set according to [1]. 

### 2.4. Gene Duplication Analyses

Inter-chromosomal gene duplications were predicted by using tBLASTn with default parameters. Each one of the 98 *VviZFP* genes were queried against *V. vinifera* chromosome sequences. Then, the tBLASTn results were parsed with Mview software [40]. Mview performs tiling of the local alignments with more than one High Scoring Pairs (HSP) (File S1, Sheet Gene_and_paralogs). A filtering criterion of the resulting alignments was applied. The filtering criteria followed: Percentage of identity over 30% and a total query coverage over 70% [41,42]. Additionally, a stricter e-value, lower than 0.00001 (instead of lower than 1.0) was considered according to [41] (Figure S1A). Paralogs on the genome were represented through a Circos plot generated using Circos software [43].

In addition, we analyzed the *VviZFP* tandem gene duplication events among *V. vinifera* chromosomes according to [44], where a chromosomal region within 200 kb containing two or more genes was defined as a tandem duplication event.

### 2.5. Calculation of dN/dS Ratio

The calculation of the dN/dS ratio was done for each pair of paralogs, one being the gene duplication found and the other being the corresponding *VviZFP* gene. The calculation was performed by the *codeml* module of PAML software [45] (File S2). Broadly, the calculation of the dN/dS ratio consists of the reason between the number of non-synonymous substitutions and the number of synonymous substitutions between a pair of messenger RNA sequences that can be conceptually translated into the same protein sequence. The parsing of the genome sequence in order to input the sequences corresponding to the mRNA paralogs to PAL2NAL was done through a Perl script with the Bioperl module Bio::DB::Fasta. The final protein sequences were manually checked using the DNA translation from Expasy (https://web.expasy.org/translate/ (accessed on 19 February 2021)) before their input to PAL2NAL. For the calculation of the dN/dS in the PAL2NAL platform [46], two files were used. The first file contained the nucleotide sequences (DNA or RNA) of the gene and the paralog while the second file had the protein translation of both (gene-paralog).

The nucleotide sequences of the paralogs were extracted from the grapevine genome with the Bioperl module Bio::DB::Fasta. After their extraction, they were translated into the protein sequence on the Expasy/translate platform and the pairs (gene-paralog) were formed manually. With both generated files, they were loaded in PAL2NAL and the dN/dS of the sequence pair was calculated (Figure S1B).

### 2.6. Gene Ontology Annotation

The functional grouping of *VviZFP* genes and the analysis of annotation data were executed with AgriGO v2 tool [47] and a singular Enrichment Analysis (SEA) using the grape transcript ID (Grape genome database) as reference was performed. We described the topmost enriched subcategories inside the first three levels of GO classification: biological processes, molecular functions and cellular components. The enrichment level of the GO term and the relative number of genes per categories are reported.

### 2.7. Expression Profiles and k-Means Clustering of VviZFP Genes Using Public Microarray Data

The *VviZFP* gene expression data was obtained from the Grape eFP Browser microarray database (http://bar.utoronto.ca/efp_grape/cgi-bin/efpWeb.cgi (accessed on 19 February 2021)) with 54 samples including green and woody tissues and organs at different developmental stages of *V. vinifera* cv Corvina [48]. The expression data obtained were normalized based on the mean expression value of each gene in all samples analyzed using *Z*-score transformation [49]. Similarity in *VviZFP* gene expression was calculated by Pearson correlation analysis and clustered by hierarchical average-linkage assay. The k-number of clusters was determined by a Figure of Merit (FOM) calculation. The data transformation, clustering and figures were generated in MultiExperiment Viewer [50].

### 2.8. Plant Material, RNA Isolation, cDNA Synthesis and Gene Expression Analysis

The plant material used in this work was obtained from a grapevine clonal plantation (*V. vinifera* cv. Carménère) kept in Los Lingues vineyard, located at Hacienda Los Lingues, Colchagua Valley, Libertador Bernardo O’higgins Region, during the 2018–2019 growing season. The sector has a Mediterranean climate, warm and sub-humid. A total of eight collections were made at different times, based on the inflorescence size from 2 cm to 10 cm.

RNA isolation was performed in three independent extractions (biological replicates) using 1–2 g of frozen tissue with the perchlorate method according to [51]. The RNAs concentration and purity were examined by measuring the optical density (OD) absorption ratio at 260 and 280 nm in a One Drop™ OD-1000 spectrophotometer (Thermo Fisher Scientific, Waltham, MA USA).

Following DNase treatment of total RNA (TURBO DNA-free Kit, Thermo Fisher Scientific), the first-strand cDNA synthesis was carried out from 2 μg of total RNA for each sample using oligo (dT) according to the manufacturer’s instructions (First Strand cDNA Synthesis Kit, Thermo Fisher Scientific, Lithuania). Gene transcript levels of *VviZFP* selected genes were measured by quantitative PCR (qPCR) using a Stratagene Mx3000P (Agilent Technologies, Santa Clara, CA, USA) system. The reactions were performed in triplicate (technical repeats) using the Maxima SYBR Green qPCR Master Mix (2×) (Thermo Scientific, USA) according to the manufacturer. The raw data were manually analyzed and normalized against *VviGADPH* expression (Locus: VIT_217s0000g10430). The primers used for qPCR analysis are listed in Table S1.

### 2.9. Promoter Analysis 

The 2000 bp upstream sequences of initiation codon from the selected *VviZFP* genes were downloaded from the *V. vinifera* cv. Carménère database [52]. The cis elements in promoter regions were identified using the PlantCARE website (http://bioinformatics.psb.ugent.be/webtools/plantcare/html/ (accessed on 19 February 2021)) [53]. Cis-element associated with hormone responses, pollen-specifc expression and others like light-responsive, CArG-box elements and TFs response elements were selected. 

### 2.10. Histochemical β-Glucuronidase (GUS) Staining Assays

For promVviZFP::GUS fusion vector construction, fragments containing a 2000 bp upstream region of *VviZFP13* and *VviZFP68* genes were amplified with a high-fidelity Platinum II Taq Hot-Start DNA Polymerase, and were cloned into the pCAMBIA1303 and pBI121 binary vectors, respectively. The constructs were introduced into *Nicotiana benthamiana* Domin leaf discs by *Agrobacterium*-mediated transformation method [54]. Flowers at different developmental stages from transgenic T1 lines were incubated with X-Gluc (5-bromo-4-chloro-3-indolyl-β-D-glucuronide) solution, cleared in 75% (*v/v*) ethanol and photographed with a stereoscope. 

## 3. Results

To identify the *VviZFP* family in *V. vinifera*, we carried out a hidden Markov model search [27] using the HMMER v3.1b2 software package. We found 101 ZFPs in the grapevine deduced proteome (Table S2) and the SMART analysis confirmed the presence of the C2H2 ZF domain in 98 of them. All members were renamed from *VviZFP1* to *VviZFP98* (See Materials and Methods).

### 3.1. General Characterization and Classification of the VviZFP Family

In general terms, the *VviZFP* family members showed high heterogeneity in most of the revised parameters. The protein lengths are diverse, VviZFP16 (74 amino acids) being the smallest and VviZFP88 (1329 amino acids) the longest amino acid sequence. The analysis of physicochemical properties showed a range of variation in molecular weight (8.9–148.4 kDa) and isoelectric point (4.2–10.3) (Table S2). Prediction of subcellular localization indicated that 96 VviZFPs could be located in the cell nucleus, consistent with previous reports about this protein group. The same analysis determined that VviZFP10 could be located in the mitochondria while VviZFP91 would be located in the extracellular space.

**Figure 1:**
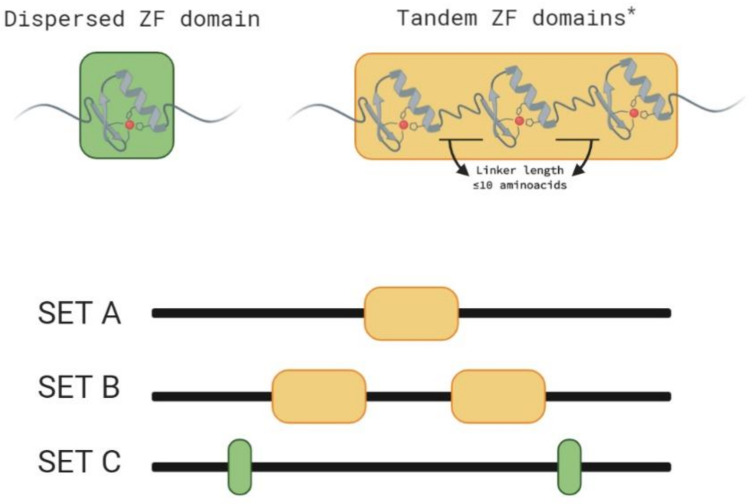
Graphical view of the classification criteria used according to ZF domain distribution. ZF arrays are composed by tandem ZFs linked by zero to ten amino acid residues, with five residues as the most frequent linker length [1]. The VviZFPs in set A possess one ZF array and the VviZFPs in set B possess more than one array of tandem ZF. Both groups may present another’s dispersed ZFs besides the ZF arrays. The VviZFPs in set C possess one or more dispersed ZFs (The ZF position showed is illustrative). * ZF arrays in grapevine contain 2 up to 4 ZF domains. Created with BioRender.com.

**Figure 2:**
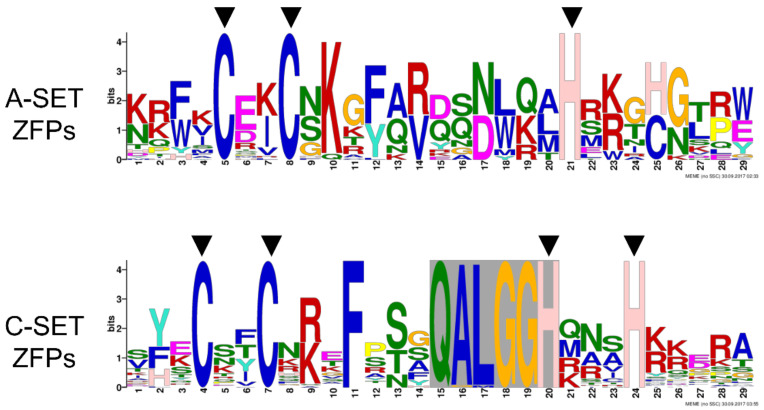
Sequence logos for the ZF motifs of VviZFPs. The x-axis represents the sequence positions in ZF domains and the y-axis represents the information content measured in bits. The sequence logos were derived from the complete peptide sequence in the MEME suite software [38].

The VviZFPs containing a tandem ZF domain in one array or in more than one array were assigned accordingly to A and B sets and the VviZFPs containing a single ZF or dispersed ZFs were assigned to the set C (Figure 1). A total of 33% of VviZFPs correspond to set A with 32 proteins that contained tandem ZF arrays, 1% corresponded to set B with one protein that contained more than one ZF array and 66% corresponded to set C with 65 proteins that contained a single or several dispersed ZFs. The VviZFPs were found to possess 1 to 9 ZF domains per protein. The sequence logo (Figure 2) shows the conservation grade among ZF motifs in the VviZFP family. In set A, the two cysteines and one histidine were largely conserved but the position of the second histidine residue was varied. In arabidopsis, the distance between the two histidines in the A1 family ZFPs varied from 3, 4, 6 or 7 residues [1]. In set C proteins, the plant-specific sequence QALGGH of ZF domains in helix position 2–7 was highly conserved. 

VviZFP52 was the only protein in the set B that has 9 ZF domains organized in three arrays through the sequence (Figure S2). VviZFP52 shared a 58.6% of protein sequence identity with arabidopsis transcription factor IIIA (TAIR code AT1G72050.2) and 66.3% with *Solanum lycopersicum* L. sequence (Solyc10g077110.1.1) suggesting that the TFIIIA homolog was also present in grapevine genome (Figure S2A). In arabidopsis, this protein contains nine ZF domains, positively regulates the transcription of 5S ribosomal RNA genes and is largely conserved between taxes [55]. BLAST searches in other plant species also revealed TFIIIA homologs in other plant genomes. Gene sequences analysis indicated that VviZFP52 contain nine C2H2 ZF domains, similar to arabidopsis TFIIIA, and other plant TFIIIA homologs (Figure S2B). Phylogenetic and sequence similarity analysis showed that all plant TFIIIAs shared a high sequence identity (Figure S2C), suggesting a conserved function in regulating the transcription of 5S RNA genes.

### 3.2. The Phylogenetic Relationships Indicate High Heterogeneity between VviZFP Family Members

**Figure 3:**
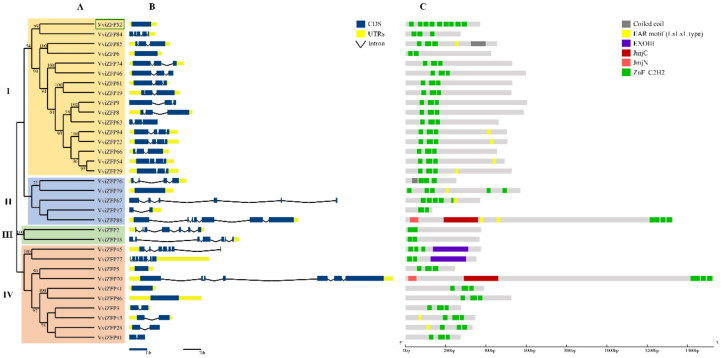
Analytical view of the set A in the *VviZFP* family. (**A**) Unrooted phylogenetic tree built by the neighbor-joining method using aligned full-length amino acid sequences. The bootstrap values lower than 50 were not shown. (**B**) Schematic diagrams for intron/exon structures of *VviZFP* genes in the grapevine genome. The blue boxes indicate the exons, the lines indicate introns and the UTRs are displayed by yellow boxes. (**C**) Schematic representation of conserved motifs within VviZFPs. Green boxes represent ZF domains and the other colored boxes indicate other conserved domains identified. Gene models, proteins and length of the motifs can be estimated using the scale at the bottom. Different groups are highlighted with colors. In green box *VviZFP52* the only B-set gene. EAR motif, Ethylene-responsive element binding factor-associated amphiphilic repression motif; EXOIII, Exonuclease RNase T/DNA polymerase III (IPR013520); JmjC, Jumonji C (IPR003347); JmjN, Jumonji N (IPR003349).

**Figure 4:**
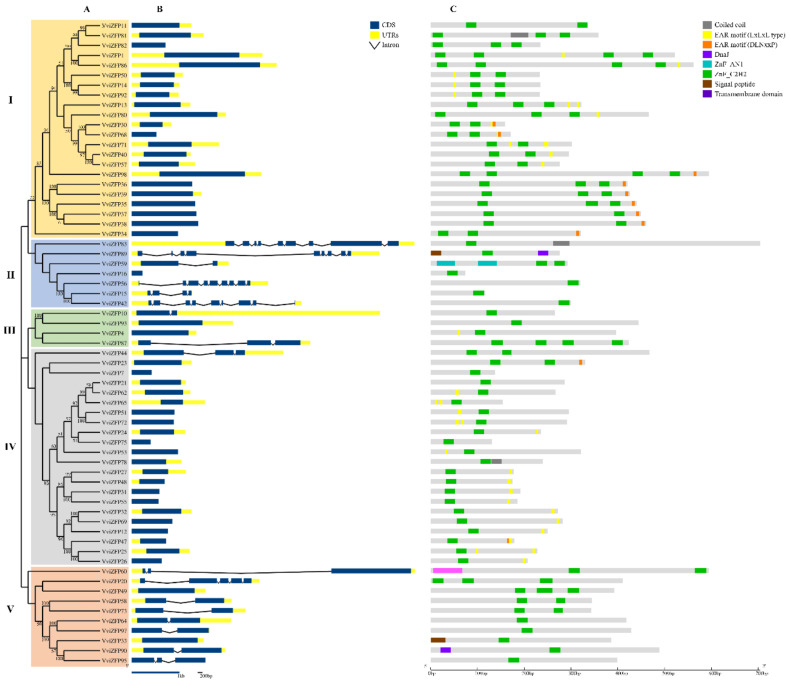
Analytical view of the set C in the *VviZFP* family. (**A**) Unrooted phylogenetic tree built by the neighbor-joining method using aligned full-length amino acid sequences. The bootstrap values lower than 50 were not shown. (**B**) Schematic diagrams for intron/exon structures of *VviZFP* genes in the grapevine genome. The blue boxes indicate the exons, the lines indicate introns and the UTRs are displayed by yellow boxes. (**C**) Schematic representation of conserved motifs within VviZFPs. Green boxes represent ZF domains and the other colored boxes indicate other conserved domains identified. Gene models, proteins and length of the motifs can be estimated using the scale at the bottom. Different groups are highlighted with colors. EAR motif, Ethylene-responsive element binding factor-associated amphiphilic repression motif (PF07897).

To evaluate the phylogenetic relationships among the VviZFP sets in grapevine, an unrooted phylogenetic tree was constructed from alignments of the amino acid sequences of A-set and C-set VviZFPs (Figure 3A and Figure 4A). The phylogenetic tree of A-set VviZFPs proteins classified the members into three major groups (I–IV) containing 15, 5, 2 and 10 proteins and the phylogenetic tree of C-set VviZFPs protein classified the members into five groups (I-V) containing 22, 7, 4, 22 and 10 proteins, respectively. The gene structure analysis determined the variable distribution of introns and exons in *VviZFP* genes. 

**Figure 5:**
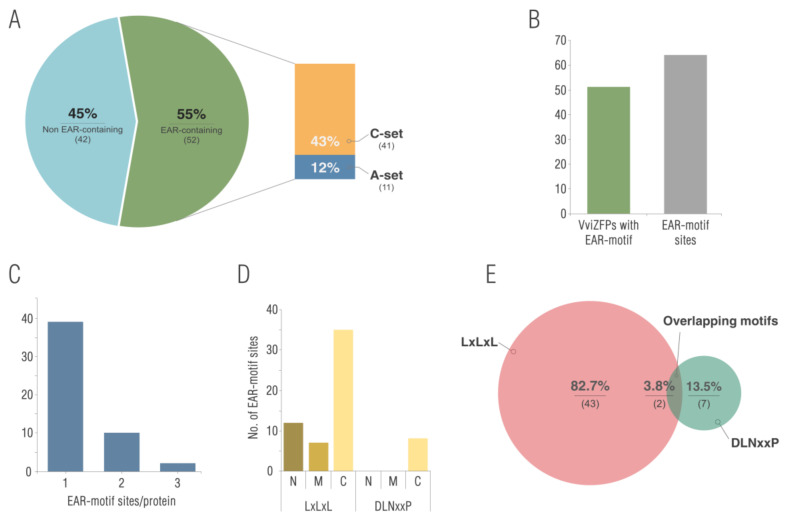
Overview of the VviZFPs EAR-repressome. (**A**) Proportion and classification of sequences containing EAR-motifs within the VviZFP family. (**B**): Total number of predicted EAR motifs containing VviZFPs, and incidence of EAR-motif sites within this protein family. (**C**): Distribution of EAR-motif sites in VviZFPs. (**D**): Frequency of EAR-motif sites in the N-terminal, C-terminal, or middle region of VviZFPs (C, C terminal; M, middle; N, N terminal). (**E**): Incidence of EAR-motif types in VviZFPs.

The gene structure indicates high heterogeneity within A-set, ranging from intron-less genes to genes with six introns (Figure 3B). This variability is observed between the groups obtained from the phylogenetic analysis. The genes of group I presents a similar gene size and mainly two introns, although genes without introns, three and four introns are also grouped together. With the exception of group III, the rest of the groups show variations in the number and length of introns. In set-C, most of the genes (74%) have zero introns (Figure 4B), and groups I and IV have genes exclusively with this genetic structure (except *VviZFP44* in group IV). On the contrary, genes with eight introns are more frequent in group II and it was not possible to determine similarities in gene structure inside group V, due to the high heterogeneity observed in the number of introns and length of the genes. VviZFP52, the only set-B protein, was aligned with A-set proteins (Figure 3) to infer about the phylogenetic relationships between both sets, based on the fact that the VviZFP52 do not have the QALGGH motif in their ZF domains, like most of the proteins in set-A (except for VviZFP79 and VviZFP5). *VviZFP52* gene does not have introns and the phylogenetic analysis determined a relationship between this gene and those belonging to group I in set A. Among 98 genes, 54 were intron-less (55%) while 14 (~15%) had 1 intron, 14 had 2 introns, 5 (5%) had 3 introns and the rest (25%) have 5 or more introns (Figure 4 and Figure 5). The gene lengths revealed variation between them. The shortest *VviZFP* gene was 226 bp (*VviZFP16*), whereas the longest one was *VviZFP70* (23.692 bp). 

### 3.3. The Domains Conserved in the VviZFPs Are Consistent with Their Role in Gene Expression Regulation

Detailed characterization of conserved protein domains between VviZFPs was conducted based on the phylogenetic trees generated. Inside set-A (Figure 3C), the group I retains a similar protein size and the position of the ZF array remains close to the N-terminus region. In the other groups (II to IV), the length of the proteins, the position of the tandem ZF domains and the presence of other protein domains vary considerably. For their part, the members in the C-set (Figure 4C) vary in the protein size, number and position of the ZF domains. Groups III and IV are characterized by having proteins with a single ZF domain (except for VviZFP87, VviZFP44 and VviZFP23) and a varied number of EAR motifs in different positions.

The sequences analysis reveals that VviZFPs conserve some domains found in proteins with previously reported roles (Figure 3C and Figure 4C). In the A-set VviZFP group, VviZFP70 a and VviZFP88 possess the Jumanji domains (JmjC and JmjN) in addition to a four-ZF-domain array (without QALGGH-motif). In arabidopsis, some histone demethylases Jumonji domain-containing proteins mediate the temporal and spatial de-repression of genes necessary for a wide range of plant processes such as hormone signaling, control of the circadian clock and flowering process [56,57,58]. Two of these proteins, EARLY FLOWERING 6 (ELF6) and RELATIVE OF EARLY FLOWERING 6 (REF6), are involved in flowering time regulation through histone modifications [56,58,59] and correspond to nuclear proteins with the same domains (C2H2 ZF and Jumanji domains) as VviZFP70 and VviZFP88. The phylogenetic analysis (Figure S3) indicates a closeness of VviZFP70 with the ELF6 proteins of woody plants and to a lesser degree with ELF6 and REF6 from arabidopsis. VviZFP88 forms another clade with the putative REF6 protein of *Vitis riparia* Michx. and is more phylogenetically distanced from the arabidopsis proteins. This analysis suggests VviZFP70 as a possible chromatin remodeler regulating the flowering time in grapevine, or even regulating other processes in this plant. In addition, the *VviZFP45* and *VviZFP77* sequences encodes to proteins with the EXOIII domain and three or two ZF in tandem, respectively. Both proteins could belong to the EXOIII family, one of the two apurinic/apyrimidinic (AP) endonucleases families involved in DNA base excision repair mechanism [60]. 

We then searched domains and protein motifs previously reported in proteins related to pollen development in order to identify potential candidates for future studies. Considering what has been reported to date, the ZFP related to pollen development may possess a nuclear localization signal (NLS), EAR-motifs and a variable number of ZF domains [11,12,14,61]. The cNLS Mapper analysis results indicate that the nuclear import of 40 members (17 in set A, 1 in set B and 22 in set C) could be mediated by its NLS and an EAR motif was identified in 49 VviZFPs (50% of total) (Figure 5). Most of these proteins correspond to C-set (39 proteins) and a less amount (10 proteins) are in A-set (Figure 5A). Furthermore, The VviZFP family predominantly possessed one EAR-motif at the C-terminus region (Figure 5C,D). Additionally, approximately 82% of these proteins contained a LxLxL type of EAR motif, 15% contained a DLNxxP type of EAR motif, and the remaining 3% had a motif where LxLxL and DLNxxP were overlapping (Figure 5E).

### 3.4. Evolution of VviZFP Gene Family

**Figure 6:**
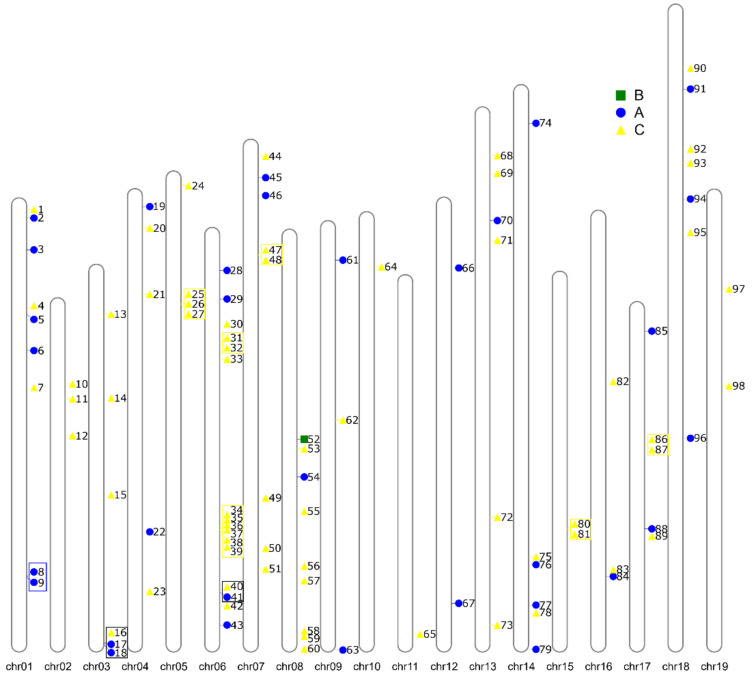
Genomic distribution and tandem duplication of *VviZFP* genes over 19 grapevine chromosomes. The chromosome number is indicated at the base of each chromosome. Each number indicates the *VviZFP* gene name and the symbol shows the position in chromosomes. Blue, green and yellow symbol correspond to set A, B and C *VviZFPs* coding genes, respectively. Tandem repeated genes are enclosed in a square.

**Figure 7:**
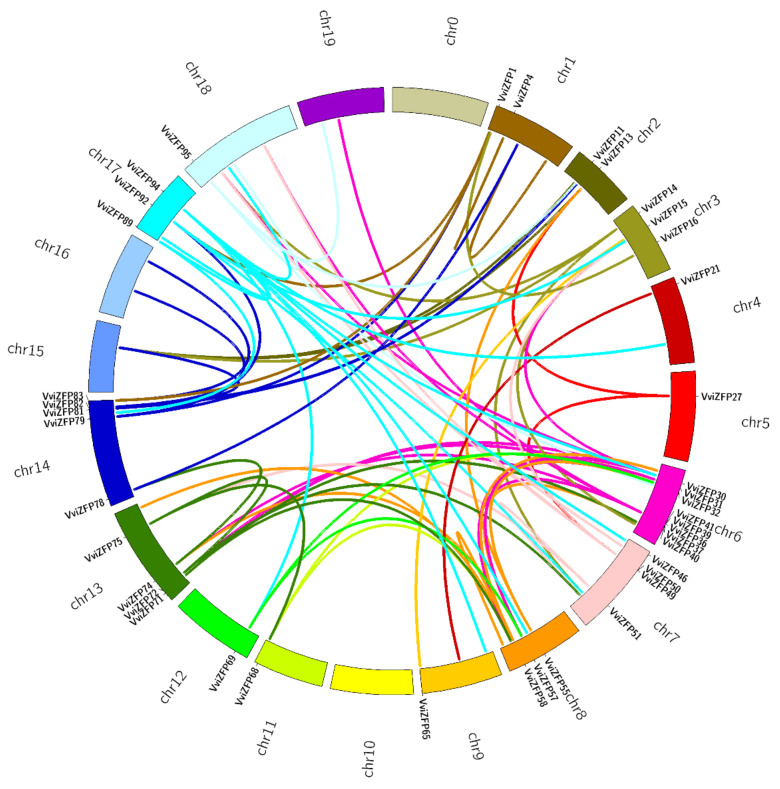
Circos plot of 41 members of *VviZFP* gene family and their paralogs along *Vitis vinifera* (assembly 12X.V2) genome. The genome is represented through its chromosomes as segments of the main circumference (Circos). The names and position of the 41 annotated *VviZFP* genes that have paralogs are labeled along the chromosomes. Inside the Circos, lines of the same color connecting two positions of the genome represent the paralog regions of each family member.

The *VviZFP* genes were widely distributed along the nineteen grapevine chromosomes (Figure 6, Table S2). These genes were more frequent in chromosome 6 containing 16 *VviZFPs* genes (0.7 genes per Mb), while chromosomes 10 and 11 contained only one *VviZFP* gene (0.04 and 0.05 genes per Mb, respectively). The average distribution of *VviZFP* genes per Mb in the grapevine genome was 0.2. Furthermore, this wide distribution could be explained in part by gene duplications generated by segmental duplication events (both intrachromosomal and interchromosomal), during molecular evolution. In this line, we identified that 24 *VviZFP* genes (File S3) were grouped into nine clusters, derived from intrachromosomal duplication event. These regions were located on chromosome 1 (one cluster), 3 (one cluster), 5 (one cluster) 6 (three clusters), 7 (one cluster), 15 (one cluster) and 17 (one cluster). The cluster located in chromosome 1 is composed by*VviZFP8* and *VviZFP9* and presents a high percentage of identity between them (67.6%). These genes are grouped together in the phylogenetic tree (In group IV of C-set), suggesting a more recent common ancestor between these two VviZFPs. Similarly, in the cluster identified in chromosome 5, a high similarity is observed between *VviZFP25* and *VviZFP26* (74.9%) and in turn a low similarity between these and *VviZFP27* (File S3). The global alignment of these sequences indicates that the ZF domain and the EAR motif are conserved in these members. The three genes are in group IV of the phylogenetic tree (Figure 4), sharing the gene structure and the arrangement of the protein motifs, which suggests that these genes come from duplication events probably at different times in the evolutionary history of the specie, being more recent the generation of *VvZFP25* and *VviZFP26* from a common ancestor. Moreover, the high frequency of *VviZFP* genes on chromosome 6 represent a rich template for genetic innovation in the grapevine genome. The first cluster in chromosome 6 is composed of *VviZFP31* and *VviZFP32*. Both proteins share a low identity percentage (33.1%) and the global alignment indicates that only the ZF domain and the EAR motif are conserved between both proteins. The phylogenetic analysis suggests a close common ancestor, both genes are grouping in group IV of C-set VviZFPs. The largest cluster is also located on chromosome 6, and is composed from *VviZFP34* to *VviZFP39*. These genes, with the exception of *VviZFP34*, are closely grouped in a clade within group I of set-C (Figure 4A). In the clade, as in the whole group, the intron-less structure is conserved (Figure 4B). The sequence similarity suggests that the formation of this gene duplications may occur via intrachromosomal duplication. The rest of the clusters show low sequence identity between the pairs, but the phylogenetic analysis groups them together (except for the cluster made up of the *VviZFP40* and *VviZFP41* genes) suggesting that they come from a common ancestor that diverged in the past. The interchromosomal duplication events are another component that could explain in part the distribution and diversity of the *VviZFP* family. Interestingly, the gene duplication analysis identified 41 paralogs apart from the 98 already mentioned. In summary, 57 *VviZFP* genes had no paralogs; 20 had one paralog; 16 could be found in duplicates; 3 could be found in triplicates; VviZFP30 had four paralogs; VviZFP94 had eight paralogs in different chromosomes (File S1, Sheet Gene_and_paralogs; Figure S4). These relationships can be visualized in the Circos plot (Figure 7). To further detect which selection process drove the evolution of the *VviZFP* gene family, we analyzed the ratios of non-synonymous (d_N_) versus synonymous (d_S_) substitution (d_N_/d_S_ ratio). A d_N_/d_S_ ratio larger than 1 is interpreted as a positive selection between the paralogs. A ratio near 1 means a neutral selection and a ratio lower than 1 is interpreted as negative, or a purifying selection between the paralogs. We observed that the d_N_/d_S_ ratio for all the paralogs was lower than 1, which suggests that the paralog sequences, in respect to their reference genes, have been under negative or purifying selection. Thus, through time, their sequences would have been selected to be translated into nearly the same protein sequence in respect to the known reference gene. A simpler alternative explanation is that these gene duplications are rather recent in grapevine evolution history. This indicates purifying selection, i.e., that the translated protein of the original gene has been maintained in the paralog genes (in the case if the paralogs are expressed and translated). A horizontal bar plot of the d_N_/d_S_ ratios for each pair of paralog genes is presented in Figure S5 and the support data in File S1, Sheet dNdS_ratio.

Then, we performed a GO-enrichment analysis to infer in relation to the biological process (BP), molecular functions (MF) and cellular component (CC) of *VviZFP* genes (Table S3, Figure S6). Most of *VviZFPs* had BP enrichment terms related to the regulation of growth and developmental processes. The MF of *VviZFP* genes included zinc ion binding, nucleic acid binding, DNA binding, protein homodimerization activity, protein dimerization activity, and transcription factor activity. The most enriched terms in CC were the nucleus and intracellular organelle, which is consistent with our cNLS Mapper analysis results.

### 3.5. Global in-silico and Real-Time Analysis of Expression Changes in the VviZFP Gene Family Shown Interesting VviZFP Candidate Genes

To visualize the global transcription profile of the *VviZFP* gene family, a heatmap was performed based on microarray data of the grapevine expression atlas (Figure S7); 13 of the 98 genes were not found in the database because the differences between the genome assembly versions used (Genoscope 12X.v0 in grapevine expression atlas and URGI 12X.v2 version used here). In general terms, some *VviZFP* genes showed similar expression profiles across the different organs/tissues evaluated, while other *VviZFPs* exhibit a specificity of expression in organs/tissues suggesting a potential functional divergence of *VviZFP* genes during the grapevine development.

For example, six *VviZFPs* (*VviZFP26*, *VviZFP47*, *VviZFP32*, *VviZFP69*, *VviZFP3*, and *VviZFP31*) were expressed at a very low level in all tested tissues, whereas *VviZFP22*, *VviZFP15* and *VviZFP42* were ubiquitously highly expressed in nearly all tissues tested. Some *VviZFPs* showed a very high level in specific organs/tissues. For example, *VviZFP46* and *VviZFP91* displayed higher expression level in seed than other organs, which suggested that they might be involved in seed development. *VviZFP24* showed relatively high expression level in tendrils young and well developed, indicating a possible participation of this gene in tendrils development. 

**Figure 8:**
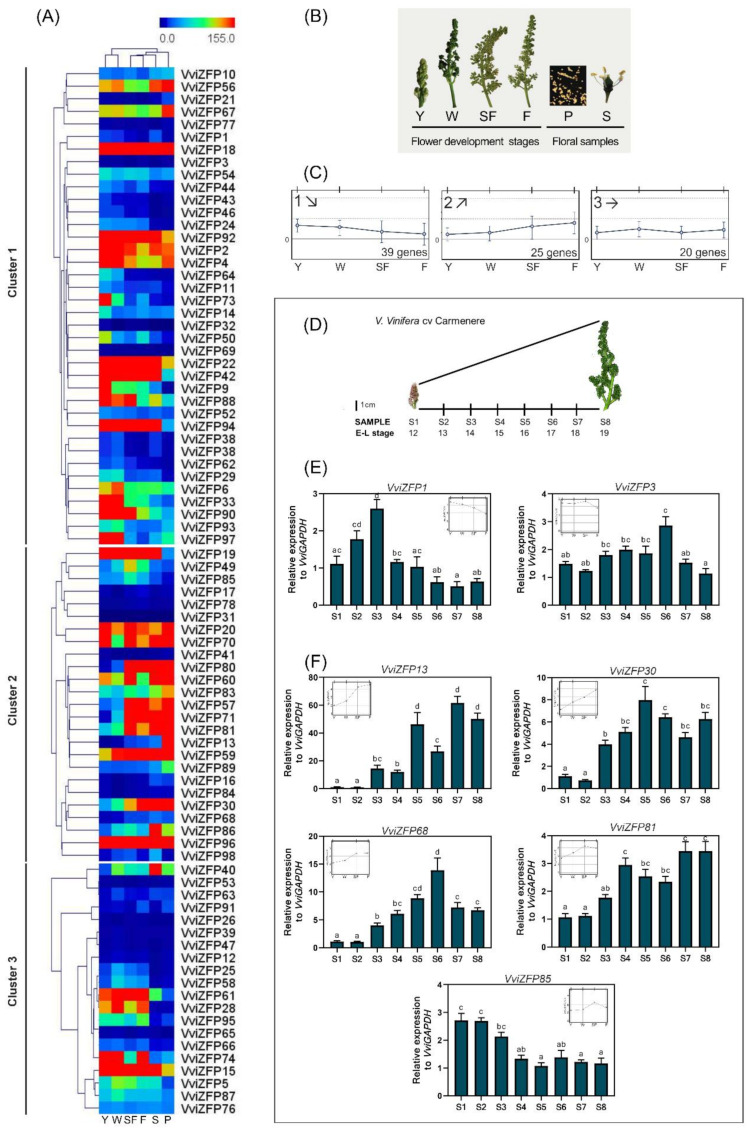
Differential expression of *VviZFP* genes over grapevine flower development. (**A**) Hierarchical clustering of the expression profiles of 85 *VviZFP* genes covering the flower development and male floral samples. (**B**) Details of samples used in the analysis. Developmental stages are abbreviated according to [48]. (**C**) Centroid graphs of *VviZFP* gene expression via k-means clustering. The patterns identified showed (1) downward and (2) upward expression along the flowering time. The (3) cluster did not show a distinguishable expression pattern. The number of genes in every cluster is indicated. (**D**–**F**) Expression profiles of *VviZFP* genes over *V. vinifera* cv. Carménère flower development. (**D**) Details of samples used in the analysis and the Eichhorn-Lorenz modified phenological stages (E–L system). The relative expression of genes selected of the (**E**) downward and (**F**) upward clusters are presented. The x-axis represents developmental stages of whole inflorescences. The bars represent the standard error of the mean (SEM) from four biological replicates. Significant differences (*p*, 0.05) of expression between samples are indicated by different letters (Tukey’s HSD test). Y, young flower; W, well developed flower; SF, start of flowering; F, flowering; P, pollen; S, stamen.

Then, to give insight into the temporal transcription patterns of *VviZFP* genes during grapevine flower development, we focused on six samples: Flower young (E–L 14), Flower-well developed (E–L 17), Start of flowering (E–L 20), Flowering (E–L 23) stamen and pollen. The information generated in the gene expression analysis serves as a preliminary basis that helps to generate subsequent hypotheses. From the heatmap (Figure 8A), contrasting expression patterns can be identified, comparing floral development with the specific expression of stamens and pollen. As an example, *VviZFP26*, *VviZFP53*, *VviZFP39*, *VviZFP47*, *VviZFP3*, *VviZFP31*, *VviZFP32* and *VviZFP69* present low levels of expression in all the samples evaluated, which suggests that these genes do not participate in flower development; on the contrary, the *VviZFP18* and *VviZFP96* genes present high levels of expression in all the samples of the analysis. *VviZFP13* is strongly expressed in pollen and, to a lesser extent, increasingly in flowers during development, which could indicate that this gene participates in the development of the male gametophyte.

The expression analysis of these stages via hierarchical and k-means clustering (*k* = 3) identified two contrasting patterns: (1) Downward (39 genes) and (2) upward (25 genes) gene expression along the flowering development (Figure 8C). The (3) pattern had 20 genes and did not show a distinguishable expression pattern. This grouping allows us to propose, based on the dynamics of ascending and descending expression throughout flower development, that within the first two groups the *VviZFP* genes that participate in pollen development could be found. Looking inside both expression clusters, we identified a group of genes that share some sequence similarity with several *C2H2 ZFPs* genes related to pollen development from arabidopsis and other species (Table S4). The gene expression of these genes was evaluated through the grapevine flower development in *V. vinifera* cv. Carménère (Figure 8D) since this is a cultivar with high tendency for PFD [19]. Our results revealed that the selected *VviZFP* genes are expressed in flowers with different specificities in terms of developmental stage. In general terms, all the *VviZFP* genes evaluated showed a dynamic expression across the flower development.

Effectively, the selected genes from the upward expression cluster, *VviZFP13*, *VviZFP30*, *VviZFP68* and *VviZFP81* had a progressive expression increase during the stages evaluated and could represent good candidates to elucidate this regulation process in grapevine. Most of the genes evaluated were consistent with the in-silico analysis, indicating similarities between both cultivars evaluated. However, cultivar-specific gene expression differences were found. An example is the *VviZFP85* pattern of gene expression in cv. Carménère were contrary to that predicted with the expression data of Corvina cultivar by the clustering analysis (Figure 8F). From the group with a downward expression pattern, the expression of *VviZFP1* and *VviZFP3* was evaluated (Figure 8E). *VviZFP1*, showed the highest expression levels during early flower development stages (S1 to S3 samples), decreasing its expression, meanwhile, in the flower development. In the in-silico analysis (Figure 8A), *VviZFP3* presents a low level of expression during floral development and no expression in pollen. Similarly, *VviZFP3* presents a low level of accumulation of transcripts during floral development in cv. Carménère, with S6 being the stage with the highest expression. The statistical analysis does not allow to determine a tendency to increase or decrease the expression of this gene.

### 3.6. The Tissue-Specific Pattern of VviZFP13 and VviZFP68 Gene Expression Suggest Their Participation in Pollen Development 

Considering the sequence similarity between *VviZFP13* and the *DAZ* genes of arabidopsis and between *VviZFP68* and the *MEZ1* gene of petunia, we wanted to determine the activity of the promoter region of both genes by generating transgenic *N. benthamiana* lines carrying a chimeric gene containing the 2000-bp upstream region of *VviZFP13* and *VviZFP68* fused with the β-glucuronidase (GUS) gene (containing the constructs promVviZFP13::GUS and promVviZFP68::GUS, respectively). 

**Figure 9:**
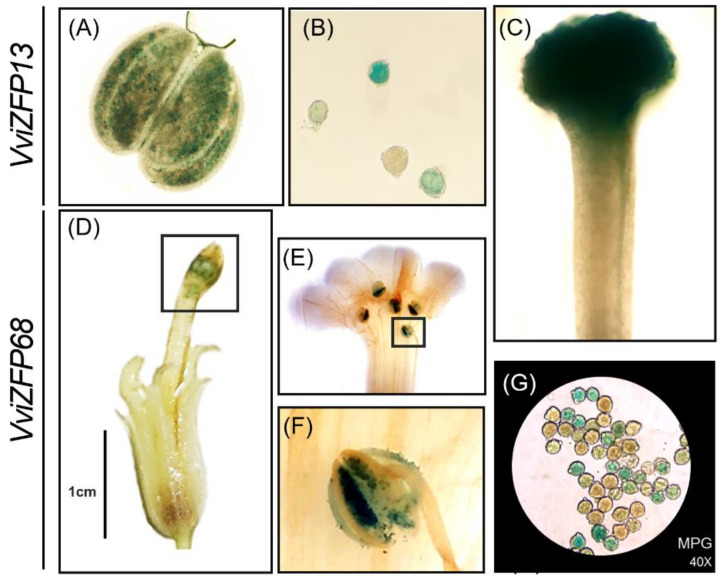
The gene expression patterns of *VviZFP13* and *VviZFP68* in flower tissues. Histochemical analysis of GUS activity in promVviZFP13::GUS and promVviZFP68::GUS transgenic *N. benthamiana* mature flowers. (**A**) mature anther; (**B**) Mature pollen grain; (**C**) Stygma and style; (**D**) whole flower; (**E**) flower open manually and (**F**) anthers close-up; (**G**) Mature pollen grains.

In promVviZFP13::GUS, strong GUS activity was detected in flowers well developed, specifically in anthers, pollen grains and stygma (Figure 9A–C). The *VviZFP13* expression in anthers is limited to male gametophyte cells (Figure 9A), which was confirmed by observing the emptied content of the anthers (Figure 9B). Similarly, the promVviZFP68::GUS lines (Figure 9D–G) exhibited GUS activity in well-developed flowers (Figure 9D), specifically in anthers (Figure 9E,F). In these organs, GUS activity is observed mainly in mature pollen grains contained in the anthers (Figure 9G). Histochemical staining assays suggests that *VviZFP13* and *VviZFP68* may be involved in development of pollen grains and are interesting candidates to characterize deeply in subsequent assays.

## 4. Discussion

In plants, the *C2H2 ZFP* gene family is the largest group of regulatory proteins and various members are known to play important roles in growth and development processes [10,62,63], hormone signal transduction [64] and stress response [65,66]. To date, genome-wide identification and characterization has been carried out in arabidopsis [1], rice [67], poplar (*Populus trichocarpa* Torr. & Gray ex Hooker) [2,68], foxtail millet (*Setaria italica* (L.) P.Beauv.) [69], maize (*Zea mays* L.) [70], tobacco (*Nicotiana tabacum* L.) [71], soybean [72], *Brassica rapa* L. [73] but has not been performed in grapevine.

We identified 98 C2H2 ZFP genes in the grapevine genome that code proteins with at least one ZF conserved motif. This number is greater than the 64-gene-list hosted in the Plant Transcription Factor Database v4.0 [74], which could mean that we identified novel variants of C2H2 ZFP genes in the grapevine genome. The *VviZFP* family size is smaller compared to arabidopsis (176), rice (189), poplar (109), foxtail millet (124), maize (211), tobacco (188) soybean (321) and *Brassica rapa* (301).

### 4.1. Insight the Evolution and Expansion of VviZFP Genes Family 

It is known that during plant and animal genome evolution, whole-genome and segmental duplication (SD) events occurred leading to an increase in biological complexity and the origin of evolutionary novelties [75,76]. Throughout grapevine evolution, significant evolutionary changes have occurred in their genome. The first characterization of the highly homozygous grapevine line PN40024 genome revealed that it contains three ancestral genomes constituting the diploid content of grapevine, being described by Jaillon [77] as a “palaeo-hexaploid” organism. An alternative explanation for the high number of triplicate regions in the grapevine genome was proposed by Velasco [78], suggesting that there was been in the *Vitis* lineage, a large-scale duplication event (probably a hybridization event), rather than before the split of *Vitis* and other dicots. These changes may have contributed to the expansion of the *VviZFP* gene family and these newly formed genes faced a choice of several fates concerning their functions [79]. Furthermore, highly similar SDs constitute templates on chromosomes for non-allelic homologous recombination (NAHR) events [80], a form of homologous recombination that occurs between two lengths of DNA that have high sequence similarity, but are not alleles. The erroneous pairing between two non-allelic SDs leads, after crossover, to, inversion, duplication, deletion or translocation rearrangements [81]. Studies about the role of SD in grapevine and other plant genomes evolution and their contribution to expansion of gene families have been published [80,81,82], revealing the large impact of SDs on the evolution of genes related to disease resistance, berry development and the ripening process [80]. Initially, duplicated genes have identical sequences and functions but tend to diverge in regulatory and coding regions. Divergence in regulatory regions can result in shifts in the expression pattern, whereas changes in the coding regions may lead to the acquisition of new functions [83].

The length and intron-exon organization in *VviZFP* genes are highly heterogeneous, implying complexity among this gene family and implying consecutive duplications and divergency events. Many duplicated *VviZFP* genes are located in collinear regions of the ancestral angiosperm genome [77]. These results are consistent with the survey of segmental and tandem duplications of ZFP genes in other model plants.

### 4.2. The Protein Domains and Motifs Identified, in Addition to the ZF, could Be Important in the VviZFPs Biological Function

The protein sequence comparison of VviZFPs reveal that almost half of the proteins could be involved in DNA transcription repression through the EAR-motif (Figure 5). This protein motif is highly conserved across evolutionarily diverse plant species and is detected in 10–25% of transcription factors belonging to multiple gene families across plants [14]. The plant family TOPLESS/TOPLESS RELATED (TPL/TPR) proteins mediate the repressor activity of different EAR-containing transcription factors by acting upon the chromatin via histone deacetylases [84,85]. In addition to ZFPs, the EAR-motif is known for its function as a negative regulator in a broad range of developmental and physiological processes such as ERF, MYB, HOMEOBOX, MADS and NAC proteins [14]. 

It has been reported that many ZFPs form a plant-specific subfamily named Q-type C2H2 zinc finger that contain a highly conserved QALGGH amino acid motif located at the N-terminus of the α-helix of the ZF domain [6]. Our phylogenetic and structural results indicate that the VviZFPs in set C correspond to Q-type C2H2 ZFPs subfamily in grapevine. The presence of domains other than the ZFs (like JmjN, JmjC or EXOIII domains) also could determine the function of grapevine *VviZFP* genes. 

### 4.3. Diverse VviZFP Genes could Be Involved in Pollen Development

Using all the information generated to gain further insights into the potential roles of the *ZFP* genes in grapevine we analyzed their spatiotemporal expression patterns during the flower development of the 7 genes selected (Figure 8). Comparing our candidates with the reported *ZFP* related to pollen development in model plants, we observed some similarities and differences. In petunia, the *PhZPT4-2* and *PhZPT4-3* genes present a major expression in the late stages of stamen development. In the grapevine genome, we identified a single gene (*VviZFP1*) as the putative homolog of these two petunia genes. The expression between the S2 and S4 stages was consistent with the moment when the pollen development occurs (data not shown), which suggests similarities in function between both species. On the other hand, unlike *PhMEZ1* in petunia (mostly expressed in anthers during early development stages), *VviZFP30* and *VviZFP68* presented a continuous and increasing expression over the flower development, preferentially expressed in S5-S6. The promoter region of *VviZFP68* is highly enriched with pollen-expression and hormone-responsive elements, as in the regulatory region of the putative ortholog *PhMEZ1* (Figure S8C,D). The *VviZFP68* promoter lacks CArG-box sites that respond to homeotic factors, unlike those found in *PhMEZ1*, which may indicate that this gene is not directly regulated by homeotic genes in grapevine. The promoter activity, specific in mature pollen grains (Figure 9D–G) could indicate that *VviZFP68* participates in the development of the male gametophyte, and integrates the regulatory network directed by homeotic genes, without being a direct target of these factors.

*VviZFP13* was the other candidate gene is since it has high sequence identity with the arabidopsis genes *AtDAZ1* and *ATDAZ2*. The gene expression of both arabidopsis genes is absent in microspores. They first appear in germ cell nuclei following microspore division, and they increase during development and persist during mature pollen [12]. In grapevine, the *VviZFP13* expression is absent at early stages, starting from S3 and moving upward from S5 to S8 (when mature pollen grains are visible). The regulatory region of *VviZFP13* is highly enriched with pollen, late pollen-expression and hormone-responsive elements (Figure S8A,B), in addition to CArG-box and AG-like elements suggesting that the regulation of this gene could be in part by homeotic factors. Added to this, the promoter region of *VviZFP13* shows activity in anthers, specifically in mature pollen grains, in a similar way to the *AtDAZ* genes expression. Additionally, it was observed that *VviZFP13* is strongly expressed in stigma, suggesting that this gene could be related to other functions in the grapevine flower development. These antecedents permit us to suggest that the *VviZFP11* gene as the putative orthologous of arabidopsis *DAZ1/DAZ2* genes and as an interesting candidate to be functionally validated in future works.

Several cellular and genetic tools, including the clustered regularly interspaced short palindromic repeats (CRISPR)/CRISPR-associated protein 9 (Cas9) system (CRISPR/Cas9 system) are available in some perennial fruit crops such as grapevine [86], and can be used to study the functions of the *VviZFP* genes in reproductive development. Since 2016, sites that are appropriate for CRISPR/Cas9-based genome editing identified in the *V. vinifera* genome are available through a publicly accessible grape CRISPR database [87]. To date, there are a growing number of reports on the use of CRISPR-Cas9 for genome editing in grapevine [88,89,90,91,92,93,94], and using it to understand the function of *VviZFP* genes can be a powerful reverse genetics approach.

The global expression of the *VviZFP* family in various tissues and stages of development (Figure S7) suggests that these genes are widely related to the regulation of several processes. In plants, a large amount of C2H2 ZFP has been reported that participate in stress and hormonal responses but due to the focus of this work have not been analyzed. As an example, *VviZFP3* is identified in a Blast analysis as the putative ortholog of arabidopsis *DEFECTIVELY ORGANIZED TRIBUTARIES 5* (*DOT5*) [95]. Seedlings with mutations in *DOT5* have a misaligned venation defect in their leaves and cotyledons in arabidopsis. *VviZFP3* and *AtDOT5* share an 68.7% of sequence identity with a high conservation of ZF domains, which suggests possible functions of a ZFP not yet characterized in grapevine.

## 5. Conclusions

In conclusion, we have identified 98 *C2H2 ZFP* genes in the grapevine genome that code proteins with at least one ZF conserved motif. The analysis allows us to infer an intricate evolution history for the *VviZFP* gene family due to the multiple paralogs recognized and the high heterogeneity in the proteins coded by these genes. We report a group of *VviZFP* genes due to the similarities with other plant ZFPs involved in pollen development regulation. According to the qPCR analysis, all the selected *VviZFP* genes present differential expression across the flower development in *V. vinifera* cv Carménère. Furthermore, GUS-based histochemical analyses performed in a heterologous system suggest the pollen-specific expression of two selected genes. Our results allow future studies to characterize *VviZFP* candidate genes related to the development of pollen in grapevine and then determine their participation in the PFD observed in different cultivars of this highly valued agronomic plant.

## Supplementary Materials

The following are available online at https://www.mdpi.com/2073-4425/12/2/302/s1, Figure S1: Diagram of Bioinformatic pipelines used. (A) Pipeline for obtaining the Circos diagram. In addition, the files with the nucleotide and amino acid sequences of the paralogs were obtained for the dN/dS calculation. (B) Pipeline for the calculation of dN/dS between pairs of sequences, of each gene and its respective paralogs, Figure S2: *VviZFP53* as the putative TFIIIA grapevine ortholog. (A) Unrooted phylogenetic tree built by the neighbor-joining method using aligned full-length amino acid sequences. (B) Schematic representation of conserved ZF domains within amino acid sequences, green boxes represent ZF domains. (C) Percent Identity Matrix between putative plants orthologs to *Arabidopsis thaliana* TFIIIA, Figure S3: Phylogenetic tree of plant ELF6 and REF6 including putative grapevine orthologs. Unrooted phylogenetic tree built by the neighbor-joining method using aligned full-length amino acid sequences, Figure S4: Histogram showing the number of paralogs that were found in the 98 genes analyzed, Figure S5: dN/dS ratio of non-synonymous (dN) to synonymous (dS) substitutions. dN/dS ratio between each gene and each of its paralogs using PAL2NAL module from PAML software, Figure S6: GO enrichment analysis of *VviZFP* gene family. The x-axis shows the enrichment score, calculated as the number of *VviZFP* genes in a given GO category divided by the total number of genes in the category. The size of the points indicates the absolute number of *VviZFP* genes in the given GO category. The color-coding indicates the adjusted p-value. Based on [96], Figure S7: The *VviZFP* gene expression atlas. Hierarchical clustering of the expression profiles of 79 *VviZFP* genes over 54 samples covering different development stages and tissues, Figure S8: Schematic view of *cis* elements in 2kb promoter region of selected *VviZFPs* and comparation with the ortholog promoter sequence. The 2kb promoter region of *VviZFP13*; (B) *AtDAZ1*; (C) *VviZFP68* and (D) *PhMEZ1*. The cis elements in promoter regions were identified using the PlantCARE tool [53]. H: hormone signaling, PS: pollen signal, O: other *cis* elements, Table S1: List of primers used in qPCR analysis, Table S2: The identified *VviZFP* genes and their information related to genomic sequence and protein product, Table S3: Significantly enriched Gene Ontology (GO) in *VviZFP* gene family, Table S4: Selected *VviZFP* genes and putative hortologs evaluated in this study, File S1: Excel File with Supplementary data information about: Sheet Gene paralogs: shows the parsed results of tBlastn of protein sequence queries against *V. vinifera* genome; Sheet Genes and paralogs: show the histogram data of gene paralogs; Sheet dN/dS results: show the output of the PAL2NAL software and the dN/dS ratio. On the Y axis, the gene, and the chromosome where it has a paralog. On the X axis the values of dN/dS ratio obtained between the gene and its paralog, File S2: Parameters to *codeml* used in pairwise comparison, File S3: Tandem duplication event regions in grapevine chromosomes. The percentage identity was obtained within the pair-wise alignment of gene sequences using Clustal Omega software [25]. The cell red intensity indicates the similarity grade between sequences. Red cells correspond to 100% sequence identity.
