# Supplementary material for: Comprehensive Genome-Wide Exploration of C2H2 Zinc Finger Family in Grapevine (Vitis vinifera L.): Insights into the Roles in the Pollen Development Regulation
Source: Genes (Basel). 2021 Feb 20;12(2):302. doi: 10.3390/genes12020302 (PMC7924211; doi:10.3390/genes12020302)
Supplement: Supplementary file 1 [file genes-12-00302-s001.zip › Files/FileS2.pdf]

Parameters to *codeml*

noisy = 0 * 0,1,2,3,9: how much rubbish on the screen
verbose = 0 * 1: detailed output, 0: concise output
runmode = -2 * 0: user tree; 1: semi-automatic; 2: automatic *3: StepwiseAddition; (4,5):PerturbationNNI; -2: pairwise

cleandata = 1 * "I added on 07/07/2004" Mikita Suyama

seqtype = 1 * 1:codons; 2:AAs; 3:codons-->AAs CodonFreq = 2 * 0:1/61 each, 1:F1X4, 2:F3X4, 3:codon table
model = 2 * models for codons: * 0:one, 1:b, 2:2 or more dN/dS ratios for branches

NSsites = 0 * dN/dS among sites. 0:no variation, 1:neutral, 2:positive
icode = 0 * 0:standard genetic code; 1:mammalian mt; 2-10:see below
Mgene = 0 * 0:rates, 1:separate; 2:pi, 3:kappa, 4:all

fix_kappa = 0 * 1: kappa fixed, 0: kappa to be estimated
kappa = 2 * initial or fixed kappa
fix_omega = 0 * 1: omega or omega_1 fixed, 0: estimate
omega = 1 * initial or fixed omega, for codons or codon-transltd AAs

fix_alpha = 1 * 0: estimate gamma shape parameter; 1: fix it at alpha
alpha = .0 * initial or fixed alpha, 0:infinity (constant rate)
Malpha = 0 * different alphas for genes
ncatG = 4 * # of categories in the dG or AdG models of rates

clock = 0 * 0: no clock, unrooted tree, 1: clock, rooted tree
getSE = 0 * 0: don't want them, 1: want S.E.s of estimates
RateAncestor = 0 * (1/0): rates (alpha>0) or ancestral states (alpha=0)
method = 0 * 0: simultaneous; 1: one branch at a time
