# Supplementary figures and images for: Comprehensive Genome-Wide Exploration of C2H2 Zinc Finger Family in Grapevine (Vitis vinifera L.): Insights into the Roles in the Pollen Development Regulation
Source: Genes (Basel). 2021 Feb 20;12(2):302. doi: 10.3390/genes12020302 (PMC7924211; doi:10.3390/genes12020302)

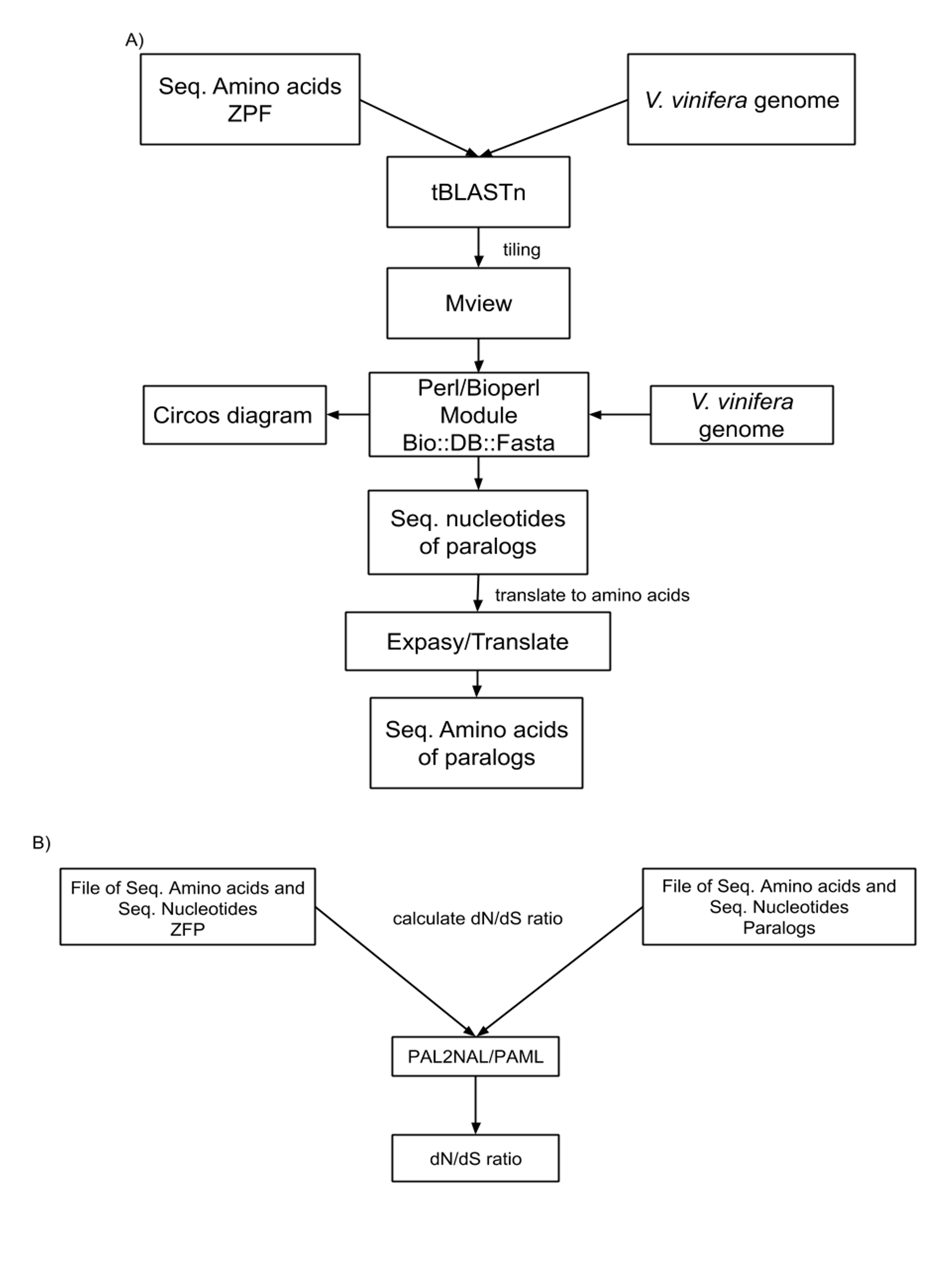

Supplement: Supplementary file 1 [file genes-12-00302-s001.zip › Figures/FigureS1.tif]

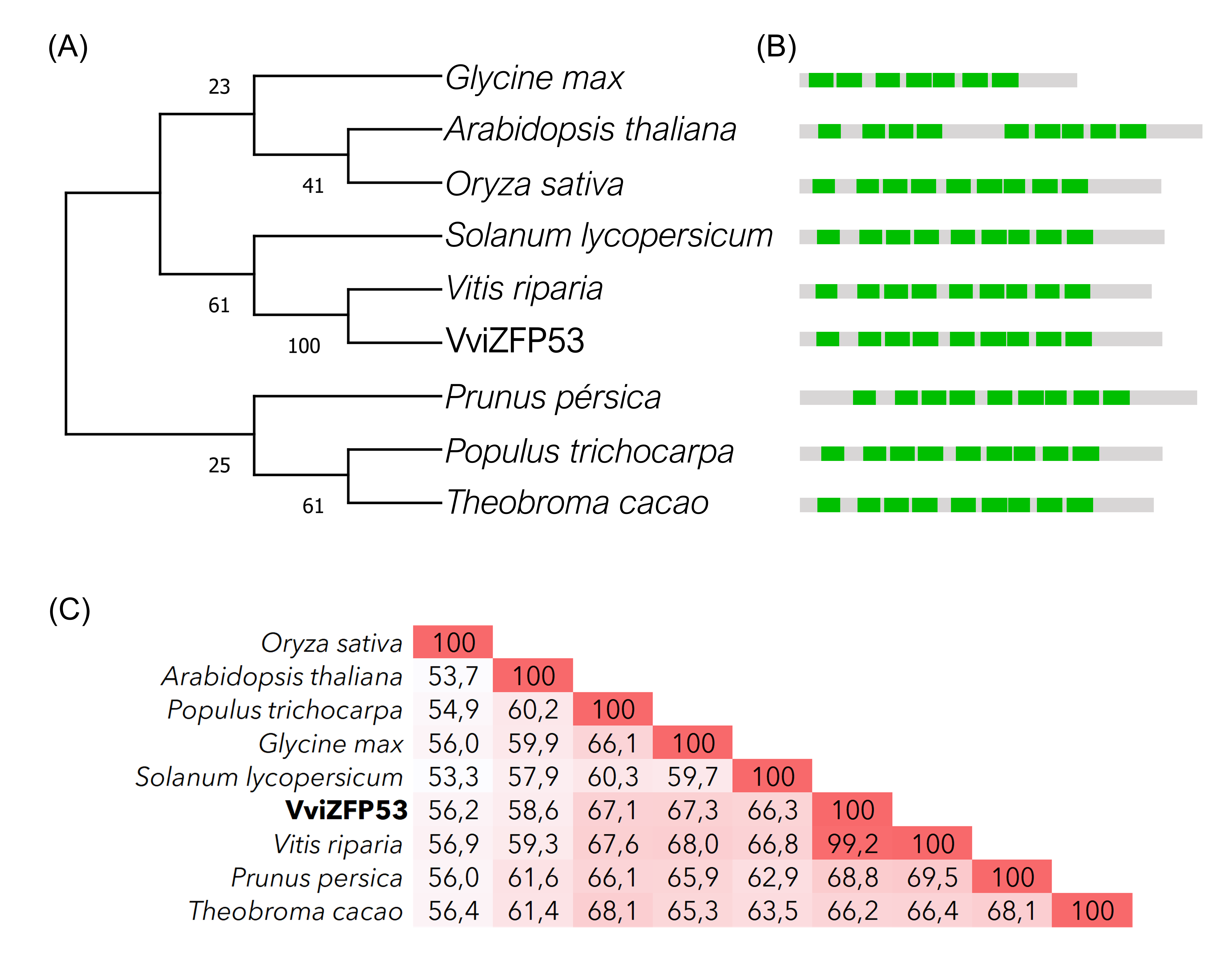

Supplement: Supplementary file 1 [file genes-12-00302-s001.zip › Figures/FigureS2.tif]

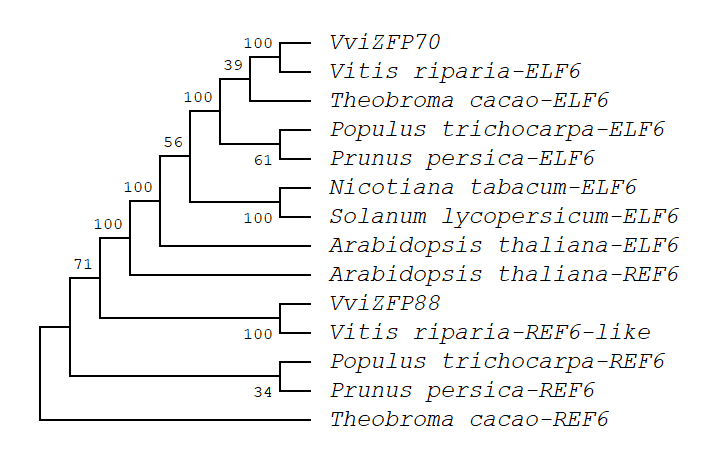

Supplement: Supplementary file 1 [file genes-12-00302-s001.zip › Figures/FigureS3.png]

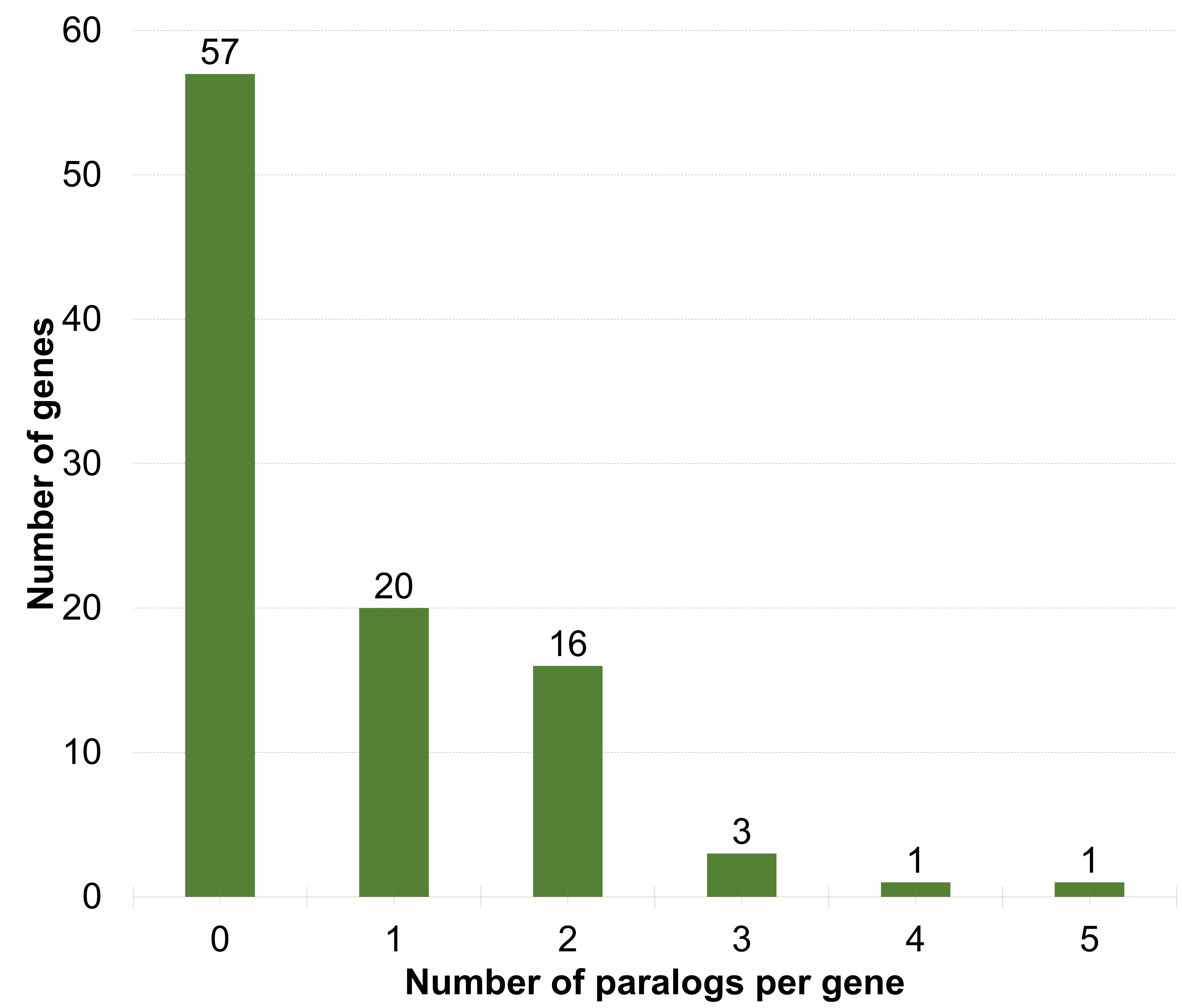

Supplement: Supplementary file 1 [file genes-12-00302-s001.zip › Figures/FigureS4.tif]

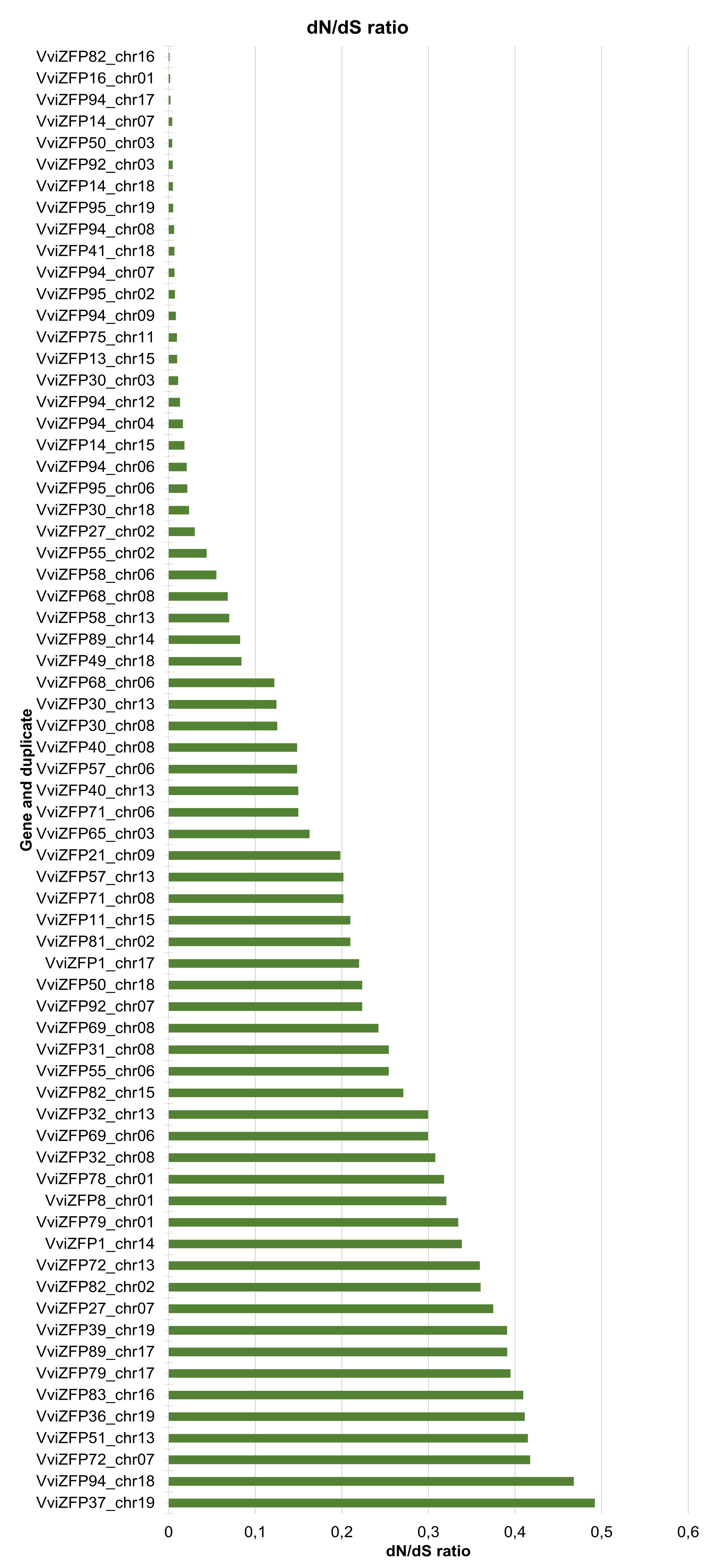

Supplement: Supplementary file 1 [file genes-12-00302-s001.zip › Figures/FigureS5.tif]

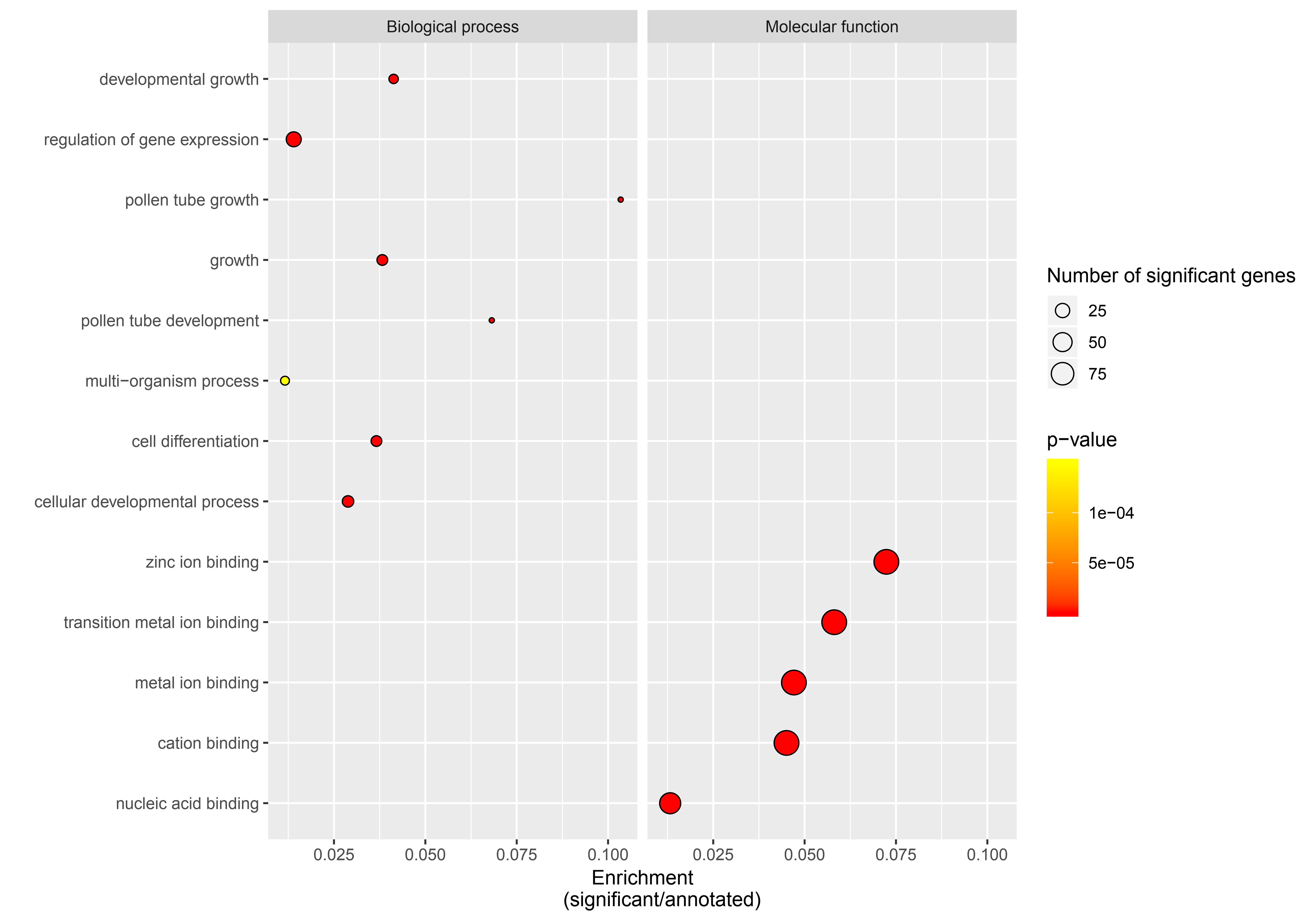

Supplement: Supplementary file 1 [file genes-12-00302-s001.zip › Figures/FigureS6.tif]

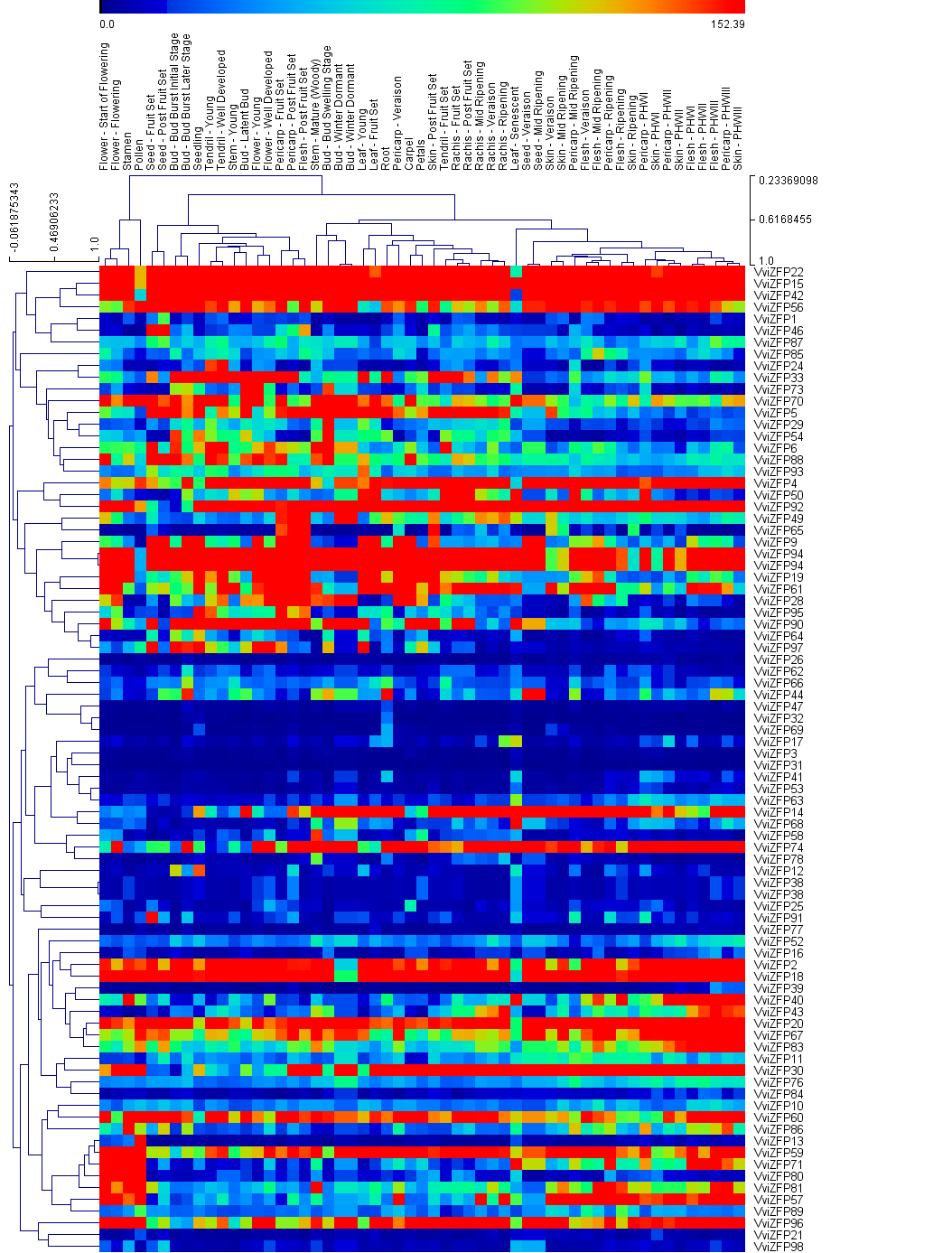

Supplement: Supplementary file 1 [file genes-12-00302-s001.zip › Figures/FigureS7.tiff]

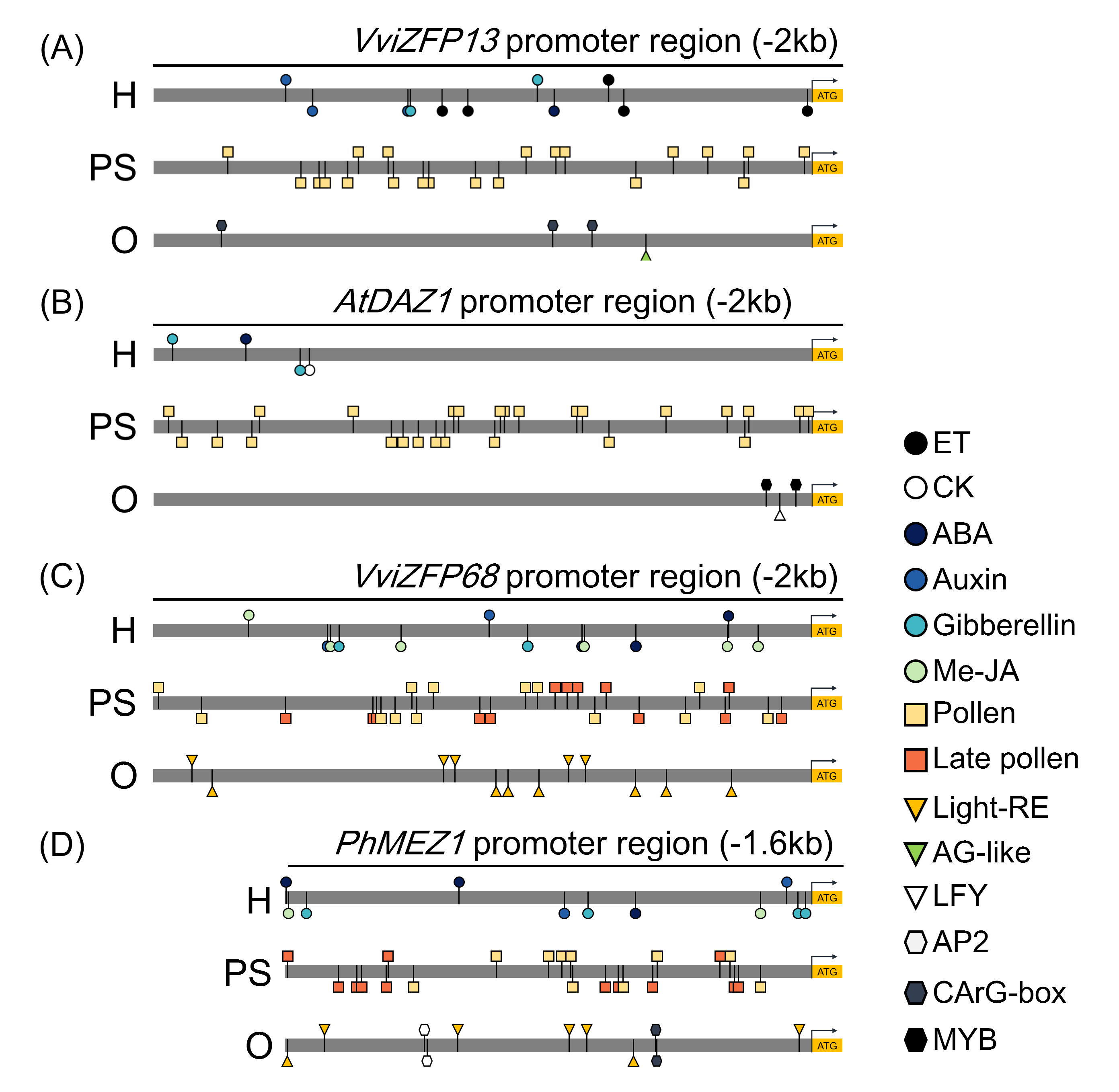

Supplement: Supplementary file 1 [file genes-12-00302-s001.zip › Figures/FigureS8.tif]
